# Crystal structure of 2-{[(*E*)-(4-anilinophen­yl)iminium­yl]meth­yl}-5-(di­ethyl­amino)­phenolate

**DOI:** 10.1107/S2056989015019490

**Published:** 2015-11-04

**Authors:** Md. Serajul Haque Faizi, Kateryna A. Ohui, Irina A. Golenya

**Affiliations:** a Department of Chemistry, Indian Institute of Technology Kanpur, Kanpur, UP 208 016, India; b National Taras Shevchenko University, Department of Chemistry, Volodymyrska str. 64, 01601 Kyiv, Ukraine

**Keywords:** crystal structure, zwitterion, *N*-phenyl-*p*-phenyl­enedi­amine, DPIM, Schiff base, hydrogen bonding

## Abstract

The title compound, C_23_H_25_N_3_O, crystallized with one single mol­ecule in the asymmetric unit and is present in the zwitterionic form. There is an intra­molecular N—H⋯O hydrogen bond in the mol­ecule with the phenol ring being inclined to the central benzene ring by 20.67 (14)°. The terminal amino­phenyl ring forms a dihedral angle of 54.21 (14)° with the central benzene ring. The two outer aromatic rings are inclined to one another by 74.54 (14)°. In the crystal, the mol­ecules are connected by N—H⋯O hydrogen bonds, with adjacent molecules related by a 2_1_ screw axis, generating *–A–B–A–B–* zigzag chains extending along [010]. The chains are linked *via* C—H⋯π and π–π inter­actions [with a centroid–centroid distance of 3.444 (3) Å] between the benzene ring and the imino group of symmetry-related mol­ecules, forming slabs lying parallel to (100).

## Abstract 2

The title compound, C_23_H_25_N_3_O, crystallized with one single mol­ecule in the asymmetric unit and present in the zwitterionic form. In the crystal, mol­ecules are connected by N—H⋯O hydrogen bonds generating *–A–B–A–B–* zigzag chains extending along [010]. The chains are linked *via* C—H⋯π inter­actions and π–π inter­actions [with a centroid–centroid distance of 3.444 (3) Å)] between the benzene ring and the imino group of symmetry-related mol­ecules, forming slabs lying parallel to (100).

## Chemical context   

Our research inter­est focuses on study of Schiff bases derived from 4-di­ethyl­amino-2-hy­droxy­benzaldehyde. It is well known that Schiff bases of salicyl­aldehyde derivative may exhibit thermochromism or photochromism, depending on the planarity or non-planarity of the mol­ecule, respectively (Cohen & Schmidt, 1964 ▸; Amimoto & Kawato, 2005 ▸). Schiff bases often exhibit various biological activities and in many cases have been shown to possess anti­bacterial, anti­cancer, anti-inflammatory and anti­toxic properties (Lozier *et al.*, 1975 ▸). They are used as anion sensors (Dalapati *et al.*, 2011 ▸), as non-linear optical compounds (Sun *et al.*, 2012 ▸) and as versatile polynuclear ligands for multinuclear magnetic exchange clusters (Moroz *et al.*, 2012 ▸). Schiff bases have also been used to prepare metal complexes (Faizi & Sen, 2014 ▸; Faizi & Hussain, 2014 ▸; Penkova *et al.*, 2010 ▸). We report herein on the crystal structure of the title compound synthesized by the condensation reaction of 4-di­ethyl­amino-2-hy­droxy­benzaldehyde and *N*-phenyl-*p*-phenyl­enedi­amine.
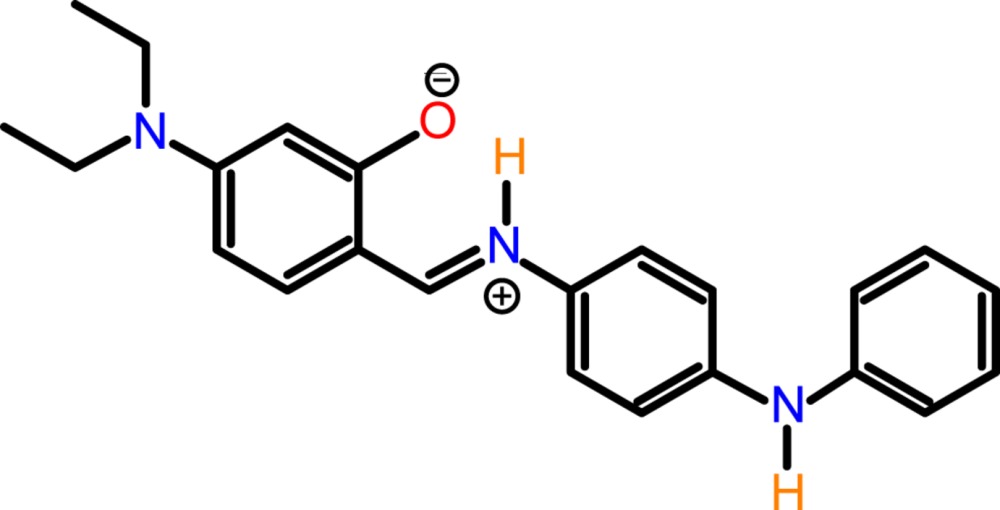


## Structural commentary   

**Figure 1:**
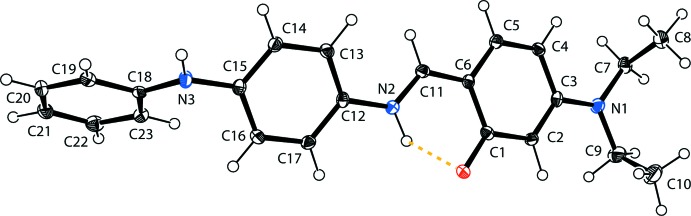
The mol­ecular structure of the title compound, showing the atom labelling and the intra­molecular N—H⋯O hydrogen bond as a dashed line (see Table 1 ▸ for details). Displacement ellipsoids are drawn at the 40% probability level.

**Figure 2:**
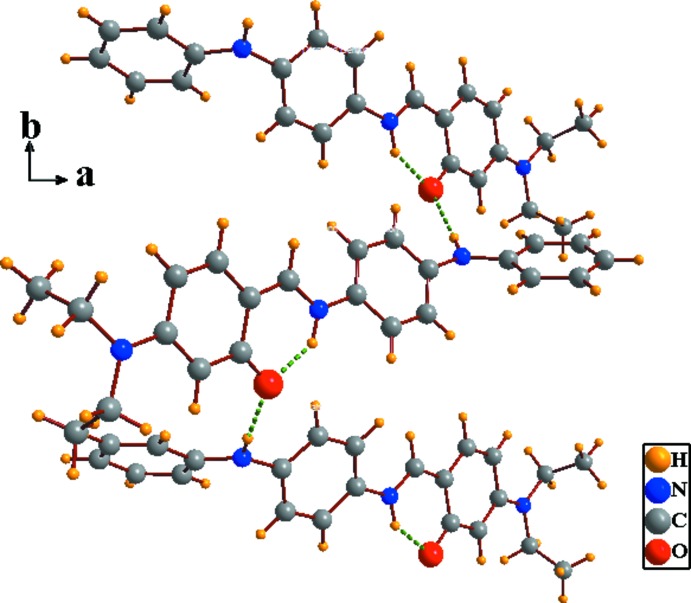
A view of the one-dimensional *–A–B–A–B–* zigzag hydrogen-bonded chain extending along the *b* axis. Hydrogen bonds are shown as dashed lines; see Table 1 ▸ for details.

In the solid state, the title compound (Fig. 1 ▸) exists in the zwitterionic form. An intra­molecular N—H⋯O hydrogen bond stabilizes the mol­ecular structure (Table 1 ▸ and Fig. 2 ▸); this is an uncommon feature in related imine-phenol compounds. The imine group, which displays a C6—C11—N2—C12 torsion angle of −178.3 (2)°, contributes to the general non-planarity of the mol­ecule. The phenol ring (C1–C6) is inclined to the central benzene ring (C12–C17) by 20.67 (14)°.

The conformation of the mol­ecule is determined by the orientation of the terminal amino­phenyl ring (C18–C23) with respect to the central benzene ring (C12–C17); the dihedral angle between them is 54.21 (14)°. The two outer aromatic rings (C18–C23 and C1–C6) are inclined to one another by 74.54 (14)°. The C—N, C=N and C—C bond lengths are normal and close to the values observed in related structures (Sliva *et al.*, 1997 ▸; Petrusenko *et al.*, 1997 ▸; Fritsky *et al.*, 2006 ▸).

## Supra­molecular features   

**Figure 3:**
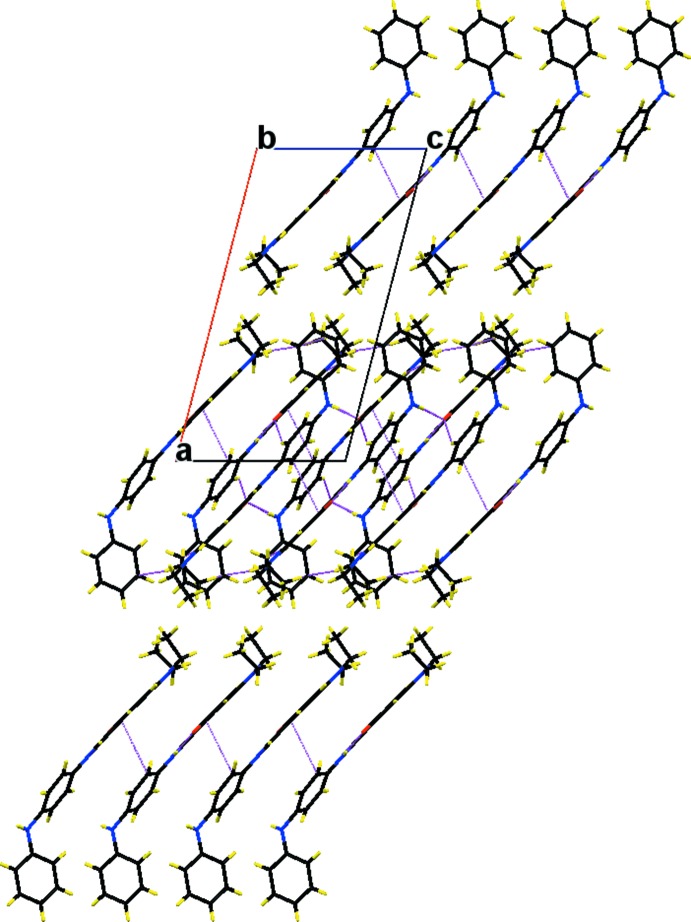
A view along the *c* axis of the crystal packing of the title compound. The hydrogen bonds, C—H⋯π inter­actions and π–π inter­actions between the benzene ring and the imino group are shown as dashed lines (see Table 1 ▸ for details; for the latter inter­actions, the atoms involved are shown).

In the crystal, mol­ecules are connected by N—H⋯O hydrogen bonds generating –*A*–*B*–*A*–*B*– zigzag chains extending along [010]; Table 1 ▸ and Fig. 3 ▸. The chains are linked *via* C—H⋯π inter­actions and π–π inter­actions between the benzene ring and the imino group of neighbouring mol­ecules, forming slabs lying parallel to (100); see Table 1 ▸ and Fig. 3 ▸. The π–π inter­actions are defined by *Cg*1⋯*Cg*2^i^ = 3.444 (3) Å, where *Cg*1 and *Cg*2 are the centroids of atoms C1–C6 and the midpoint of atoms N2/C11, respectively [symmetry code: (i) *x*, −*y* + 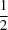, *z* − 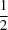].

## Database survey   

There are very few examples of similar compounds in the literature although some metal complexes of similar ligands have been reported (Xie *et al.*, 2013 ▸; Safin *et al.*, 2012 ▸). A search of the Cambridge Structural Database (Version 5.35, May 2014; Groom & Allen, 2014 ▸) revealed the structure of one very similar compound, *viz. N*-[(*E*)-4-chloro­benzyl­idene]-*N*′-phenyl­benzene-1,4-di­amine (Nor Hashim *et al.*, 2010 ▸, in which the 2-phenol ring in the title compound is replaced by a 4-chloro­benzene ring. The central six-membered ring makes a dihedral angle of 12.26 (10)° with the 4-chloro­phenyl ring. The corresponding dihedral angle in the title compound is 20.67 (14)°.

## Synthesis and crystallization   

100 mg (1 mmol) of *N*-phenyl-*p*-phenyl­enedi­amine was dissolved in 10 ml of absolute ethanol. To this solution, 85 mg (1 mmol) of 4-di­ethyl­amino-2-hy­droxy­benzaldehyde in 5 ml of absolute ethanol was dropwisely added under stirring. This mixture was stirred for 10 min, two drops of glacial acetic acid were then added and the mixture was further refluxed for 2 h. The resulting yellow precipitate was recovered by filtration, washed several times with a small portions of EtOH and then with diethyl ether to give 150 mg (88%) of 5-di­ethyl­amino-2-[(*E*)-{[4-(phenyl­amino)­phen­yl]imino­meth­yl}phenol] (DPIM). Crystals of the title compound suitable for X-ray analysis were obtained within three days by slow evaporation of the DMF solvent.

## Refinement   

Crystal data, data collection and structure refinement details are summarized in Table 2 ▸. The N—H and H atoms were located in a difference Fourier map. Their positional and isotropic thermal parameters were included in further stages of the refinement. All C-bound H atoms were positioned geometrically and refined using a riding model with C—H = 0.93–0.97 Å and with *U*_iso_(H) = 1.2–1.5*U*_eq_(C).

## Supplementary Material

Crystal structure: contains datablock(s) global, I. DOI: 10.1107/S2056989015019490/lh5793sup1.cif

Structure factors: contains datablock(s) I. DOI: 10.1107/S2056989015019490/lh5793Isup2.hkl

Supporting information file. DOI: 10.1107/S2056989015019490/lh5793Isup3.cml

CCDC reference: 1431311

Additional supporting information:  crystallographic information; 3D view; checkCIF report

## Figures and Tables

**Table 1:** Hydrogen-bond geometry (Å, °) *Cg* is the centroid of the C1–C6 ring.

*D*—H⋯*A*	*D*—H	H⋯*A*	*D*⋯*A*	*D*—H⋯*A*
N2—H2*N*⋯O1	0.90 (2)	1.83 (2)	2.609 (2)	143 (2)
N3—H3*H*⋯O1^i^	0.85 (2)	2.05 (2)	2.900 (3)	175 (2)
C7—H7*A*⋯*Cg* ^ii^	0.97	2.87	3.465 (3)	121

Symmetry codes: (i) 
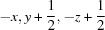
; (ii) 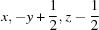.

**Table 2:** Experimental details

Crystal data	
Chemical formula	C_23_H_25_N_3_O
*M*_r_	359.46
Crystal system, space group	Monoclinic, *P*2_1_/*c*
Temperature (K)	100
*a*, *b*, *c* (Å)	18.0358 (16), 11.3851 (8), 9.4815 (9)
β (°)	104.560 (3)
*V* (Å^3^)	1884.4 (3)
*Z*	4
Radiation type	Mo *K*α
μ (mm^−1^)	0.08
Crystal size (mm)	0.20 × 0.15 × 0.12

Data collection	
Diffractometer	Bruker SMART APEX CCD
Absorption correction	Multi-scan (*SADABS*; Sheldrick, 2004 ▸)
*T*_min_, *T*_max_	0.984, 0.991
No. of measured, independent and observed [*I* > 2σ(*I*)’] reflections	14768, 3322, 2186
*R*_int_	0.078
(sin θ/λ)_max_ (Å^−1^)	0.595

Refinement	
*R*[*F* ^2^ > 2σ(*F* ^2^)], *wR*(*F* ^2^), *S*	0.054, 0.138, 1.00
No. of reflections	3322
No. of parameters	254
H-atom treatment	H atoms treated by a mixture of independent and constrained refinement
Δρ_max_, Δρ_min_ (e Å^−3^)	0.24, −0.30

Computer programs: *SMART* and *SAINT* (Bruker, 2003 ▸), *SIR97* (Altomare *et al.*, 1999 ▸), *SHELXL97* (Sheldrick, 2008 ▸), *DIAMOND* (Brandenberg & Putz, 2006 ▸), *Mercury* (Macrae *et al.*, 2008 ▸) and *PLATON* (Spek, 2009 ▸).

## supplementary crystallographic information

### Crystal data

**Table:**

C_23_H_25_N_3_O	*F*(000) = 768
*M**_r_* = 359.46	*D*_x_ = 1.267 Mg m^−^^3^
Monoclinic, *P*2_1_/*c*	Melting point: 270 K
Hall symbol: -P 2ybc	Mo *K*α radiation, λ = 0.71073 Å
*a* = 18.0358 (16) Å	Cell parameters from 2553 reflections
*b* = 11.3851 (8) Å	θ = 2.7–23.7°
*c* = 9.4815 (9) Å	µ = 0.08 mm^−^^1^
β = 104.560 (3)°	*T* = 100 K
*V* = 1884.4 (3) Å^3^	Needle, dark yellow
*Z* = 4	0.20 × 0.15 × 0.12 mm

### Data collection

**Table:**

Bruker SMART APEX CCD diffractometer	3322 independent reflections
Radiation source: fine-focus sealed tube	2186 reflections with *I* > 2σ(*I*)'
Graphite monochromator	*R*_int_ = 0.078
ω–scans	θ_max_ = 25.0°, θ_min_ = 2.9°
Absorption correction: multi-scan (*SADABS*; Sheldrick, 2004)	*h* = −19→21
*T*_min_ = 0.984, *T*_max_ = 0.991	*k* = −13→13
14768 measured reflections	*l* = −11→11

### Refinement

**Table:**

Refinement on *F*^2^	Primary atom site location: structure-invariant direct methods
Least-squares matrix: full	Secondary atom site location: difference Fourier map
*R*[*F*^2^ > 2σ(*F*^2^)] = 0.054	Hydrogen site location: inferred from neighbouring sites
*wR*(*F*^2^) = 0.138	H atoms treated by a mixture of independent and constrained refinement
*S* = 1.00	*w* = 1/[σ^2^(*F*_o_^2^) + (0.0763*P*)^2^] where *P* = (*F*_o_^2^ + 2*F*_c_^2^)/3
3322 reflections	(Δ/σ)_max_ < 0.001
254 parameters	Δρ_max_ = 0.24 e Å^−^^3^
0 restraints	Δρ_min_ = −0.30 e Å^−^^3^

### Special details

**Table:**

Geometry. All esds (except the esd in the dihedral angle between two l.s. planes) are estimated using the full covariance matrix. The cell esds are taken into account individually in the estimation of esds in distances, angles and torsion angles; correlations between esds in cell parameters are only used when they are defined by crystal symmetry. An approximate (isotropic) treatment of cell esds is used for estimating esds involving l.s. planes.
Refinement. Refinement of F^2^ against ALL reflections. The weighted R-factor wR and goodness of fit S are based on F^2^, conventional R-factors R are based on F, with F set to zero for negative F^2^. The threshold expression of F^2^ > 2sigma(F^2^) is used only for calculating R-factors(gt) etc. and is not relevant to the choice of reflections for refinement. R-factors based on F^2^ are statistically about twice as large as those based on F, and R- factors based on ALL data will be even larger.

### Fractional atomic coordinates and isotropic or equivalent isotropic displacement parameters (Å^2^)

**Table:**

	*x*	*y*	*z*	*U*_iso_*/*U*_eq_	
C1	0.16973 (12)	0.14061 (19)	−0.0731 (3)	0.0162 (5)	
C2	0.22412 (11)	0.10711 (19)	−0.1497 (2)	0.0168 (5)	
H2	0.2319	0.0275	−0.1624	0.020*	
C3	0.26674 (11)	0.18820 (19)	−0.2072 (2)	0.0160 (5)	
C4	0.25415 (12)	0.31118 (19)	−0.1877 (3)	0.0174 (5)	
H4	0.2823	0.3671	−0.2235	0.021*	
C5	0.20136 (12)	0.34542 (19)	−0.1173 (3)	0.0185 (5)	
H5	0.1937	0.4254	−0.1069	0.022*	
C6	0.15719 (11)	0.26528 (18)	−0.0586 (2)	0.0156 (5)	
C7	0.36691 (12)	0.2388 (2)	−0.3332 (3)	0.0220 (6)	
H7A	0.3355	0.3049	−0.3767	0.026*	
H7B	0.3863	0.2018	−0.4089	0.026*	
C8	0.43420 (13)	0.2845 (2)	−0.2161 (3)	0.0284 (6)	
H8A	0.4157	0.3308	−0.1476	0.043*	
H8B	0.4661	0.3322	−0.2602	0.043*	
H8C	0.4634	0.2195	−0.1666	0.043*	
C9	0.33326 (15)	0.0308 (2)	−0.3032 (3)	0.0325 (7)	
H9A	0.3523	0.0229	−0.3898	0.039*	
H9B	0.2852	−0.0117	−0.3203	0.039*	
C10	0.39020 (15)	−0.0248 (2)	−0.1758 (3)	0.0445 (8)	
H10A	0.4397	0.0102	−0.1658	0.067*	
H10B	0.3932	−0.1076	−0.1927	0.067*	
H10C	0.3738	−0.0122	−0.0882	0.067*	
C11	0.10234 (11)	0.30625 (19)	0.0085 (2)	0.0175 (5)	
H11	0.0970	0.3871	0.0163	0.021*	
C12	−0.00088 (11)	0.27358 (19)	0.1280 (2)	0.0159 (5)	
C13	−0.00286 (12)	0.38568 (19)	0.1858 (3)	0.0180 (6)	
H13	0.0362	0.4388	0.1853	0.022*	
C14	−0.06264 (11)	0.41801 (19)	0.2439 (3)	0.0183 (6)	
H14	−0.0639	0.4936	0.2805	0.022*	
C15	−0.12107 (12)	0.33966 (19)	0.2488 (3)	0.0163 (5)	
C16	−0.11642 (12)	0.22592 (19)	0.1981 (3)	0.0202 (6)	
H16	−0.1534	0.1710	0.2052	0.024*	
C17	−0.05756 (12)	0.19375 (19)	0.1374 (3)	0.0182 (5)	
H17	−0.0558	0.1178	0.1024	0.022*	
C18	−0.25825 (12)	0.36660 (18)	0.2449 (3)	0.0167 (5)	
C19	−0.30949 (12)	0.41195 (19)	0.3197 (3)	0.0201 (6)	
H19	−0.2908	0.4431	0.4126	0.024*	
C20	−0.38707 (12)	0.41113 (19)	0.2581 (3)	0.0231 (6)	
H20	−0.4201	0.4423	0.3095	0.028*	
C21	−0.41679 (13)	0.3645 (2)	0.1205 (3)	0.0251 (6)	
H21	−0.4693	0.3646	0.0789	0.030*	
C22	−0.36671 (13)	0.31798 (19)	0.0465 (3)	0.0241 (6)	
H22	−0.3860	0.2859	−0.0457	0.029*	
C23	−0.28811 (12)	0.31815 (19)	0.1069 (3)	0.0204 (6)	
H23	−0.2554	0.2860	0.0555	0.025*	
N1	0.31894 (10)	0.15485 (16)	−0.2813 (2)	0.0205 (5)	
N2	0.05736 (10)	0.23777 (17)	0.0618 (2)	0.0170 (5)	
N3	−0.18057 (10)	0.37535 (18)	0.3112 (2)	0.0199 (5)	
O1	0.13165 (8)	0.06417 (12)	−0.01909 (17)	0.0187 (4)	
H2N	0.0678 (14)	0.161 (2)	0.050 (3)	0.038 (8)*	
H3H	−0.1685 (13)	0.430 (2)	0.374 (3)	0.024 (7)*	

### Atomic displacement parameters (Å^2^)

**Table:**

	*U*^11^	*U*^22^	*U*^33^	*U*^12^	*U*^13^	*U*^23^
C1	0.0140 (11)	0.0188 (12)	0.0139 (14)	−0.0018 (10)	−0.0001 (10)	0.0019 (10)
C2	0.0178 (12)	0.0127 (12)	0.0190 (15)	0.0013 (9)	0.0028 (11)	−0.0016 (10)
C3	0.0122 (11)	0.0231 (13)	0.0108 (13)	0.0026 (10)	−0.0006 (10)	−0.0003 (10)
C4	0.0147 (12)	0.0174 (12)	0.0191 (14)	−0.0012 (10)	0.0026 (11)	0.0042 (10)
C5	0.0168 (12)	0.0165 (12)	0.0204 (15)	0.0024 (10)	0.0012 (11)	0.0018 (10)
C6	0.0119 (11)	0.0172 (12)	0.0161 (14)	0.0006 (10)	0.0008 (10)	0.0015 (10)
C7	0.0190 (12)	0.0279 (14)	0.0209 (15)	0.0008 (11)	0.0086 (11)	0.0013 (11)
C8	0.0212 (13)	0.0314 (15)	0.0331 (17)	−0.0008 (11)	0.0074 (12)	0.0004 (12)
C9	0.0409 (15)	0.0240 (14)	0.0423 (19)	0.0041 (12)	0.0286 (14)	−0.0026 (13)
C10	0.0403 (16)	0.0339 (16)	0.069 (2)	0.0145 (13)	0.0320 (17)	0.0186 (16)
C11	0.0166 (12)	0.0146 (12)	0.0191 (15)	−0.0008 (10)	0.0004 (11)	0.0022 (10)
C12	0.0134 (11)	0.0201 (13)	0.0138 (14)	0.0018 (10)	0.0024 (10)	0.0015 (10)
C13	0.0139 (12)	0.0185 (13)	0.0200 (15)	−0.0027 (10)	0.0015 (11)	0.0003 (10)
C14	0.0166 (12)	0.0172 (12)	0.0200 (15)	0.0010 (10)	0.0026 (11)	−0.0029 (10)
C15	0.0144 (11)	0.0195 (13)	0.0142 (14)	0.0019 (10)	0.0021 (10)	0.0000 (10)
C16	0.0164 (12)	0.0177 (13)	0.0264 (16)	−0.0027 (10)	0.0054 (11)	0.0016 (11)
C17	0.0173 (12)	0.0152 (12)	0.0206 (15)	0.0005 (10)	0.0021 (11)	−0.0027 (10)
C18	0.0161 (12)	0.0121 (11)	0.0222 (15)	0.0000 (10)	0.0054 (11)	0.0052 (10)
C19	0.0207 (12)	0.0173 (13)	0.0232 (15)	−0.0022 (10)	0.0073 (11)	−0.0002 (11)
C20	0.0174 (13)	0.0212 (13)	0.0336 (18)	0.0009 (10)	0.0120 (12)	0.0020 (12)
C21	0.0134 (12)	0.0228 (13)	0.0367 (18)	−0.0020 (10)	0.0019 (12)	0.0015 (12)
C22	0.0227 (13)	0.0225 (13)	0.0238 (16)	−0.0027 (11)	−0.0003 (12)	−0.0014 (11)
C23	0.0198 (12)	0.0176 (13)	0.0246 (16)	0.0018 (10)	0.0069 (11)	0.0012 (11)
N1	0.0204 (10)	0.0201 (11)	0.0232 (13)	0.0025 (9)	0.0095 (9)	0.0008 (9)
N2	0.0165 (10)	0.0148 (11)	0.0202 (13)	0.0016 (9)	0.0056 (9)	0.0002 (9)
N3	0.0138 (10)	0.0225 (12)	0.0238 (13)	−0.0010 (9)	0.0052 (9)	−0.0077 (10)
O1	0.0161 (8)	0.0164 (8)	0.0241 (10)	−0.0014 (7)	0.0064 (7)	0.0021 (7)

### Geometric parameters (Å, º)

**Table:**

C1—O1	1.292 (2)	C11—H11	0.9300
C1—C2	1.412 (3)	C12—C17	1.387 (3)
C1—C6	1.449 (3)	C12—C13	1.393 (3)
C2—C3	1.397 (3)	C12—N2	1.413 (3)
C2—H2	0.9300	C13—C14	1.378 (3)
C3—N1	1.363 (3)	C13—H13	0.9300
C3—C4	1.438 (3)	C14—C15	1.390 (3)
C4—C5	1.351 (3)	C14—H14	0.9300
C4—H4	0.9300	C15—C16	1.391 (3)
C5—C6	1.414 (3)	C15—N3	1.409 (3)
C5—H5	0.9300	C16—C17	1.378 (3)
C6—C11	1.385 (3)	C16—H16	0.9300
C7—N1	1.455 (3)	C17—H17	0.9300
C7—C8	1.516 (3)	C18—N3	1.388 (3)
C7—H7A	0.9700	C18—C23	1.397 (3)
C7—H7B	0.9700	C18—C19	1.398 (3)
C8—H8A	0.9600	C19—C20	1.374 (3)
C8—H8B	0.9600	C19—H19	0.9300
C8—H8C	0.9600	C20—C21	1.385 (3)
C9—N1	1.460 (3)	C20—H20	0.9300
C9—C10	1.513 (4)	C21—C22	1.381 (3)
C9—H9A	0.9700	C21—H21	0.9300
C9—H9B	0.9700	C22—C23	1.389 (3)
C10—H10A	0.9600	C22—H22	0.9300
C10—H10B	0.9600	C23—H23	0.9300
C10—H10C	0.9600	N2—H2N	0.90 (2)
C11—N2	1.314 (3)	N3—H3H	0.85 (2)
			
O1—C1—C2	122.0 (2)	C17—C12—C13	118.9 (2)
O1—C1—C6	120.73 (19)	C17—C12—N2	118.80 (19)
C2—C1—C6	117.29 (19)	C13—C12—N2	122.34 (19)
C3—C2—C1	123.0 (2)	C14—C13—C12	120.1 (2)
C3—C2—H2	118.5	C14—C13—H13	119.9
C1—C2—H2	118.5	C12—C13—H13	119.9
N1—C3—C2	122.4 (2)	C13—C14—C15	121.2 (2)
N1—C3—C4	119.32 (19)	C13—C14—H14	119.4
C2—C3—C4	118.23 (19)	C15—C14—H14	119.4
C5—C4—C3	119.9 (2)	C14—C15—C16	118.27 (19)
C5—C4—H4	120.0	C14—C15—N3	119.5 (2)
C3—C4—H4	120.0	C16—C15—N3	122.1 (2)
C4—C5—C6	123.0 (2)	C17—C16—C15	120.8 (2)
C4—C5—H5	118.5	C17—C16—H16	119.6
C6—C5—H5	118.5	C15—C16—H16	119.6
C11—C6—C5	120.1 (2)	C16—C17—C12	120.7 (2)
C11—C6—C1	121.3 (2)	C16—C17—H17	119.7
C5—C6—C1	118.54 (19)	C12—C17—H17	119.7
N1—C7—C8	114.40 (19)	N3—C18—C23	124.1 (2)
N1—C7—H7A	108.7	N3—C18—C19	117.7 (2)
C8—C7—H7A	108.7	C23—C18—C19	118.2 (2)
N1—C7—H7B	108.7	C20—C19—C18	120.9 (2)
C8—C7—H7B	108.7	C20—C19—H19	119.5
H7A—C7—H7B	107.6	C18—C19—H19	119.5
C7—C8—H8A	109.5	C19—C20—C21	121.0 (2)
C7—C8—H8B	109.5	C19—C20—H20	119.5
H8A—C8—H8B	109.5	C21—C20—H20	119.5
C7—C8—H8C	109.5	C22—C21—C20	118.5 (2)
H8A—C8—H8C	109.5	C22—C21—H21	120.7
H8B—C8—H8C	109.5	C20—C21—H21	120.7
N1—C9—C10	113.6 (2)	C21—C22—C23	121.3 (2)
N1—C9—H9A	108.9	C21—C22—H22	119.3
C10—C9—H9A	108.9	C23—C22—H22	119.3
N1—C9—H9B	108.9	C22—C23—C18	120.0 (2)
C10—C9—H9B	108.9	C22—C23—H23	120.0
H9A—C9—H9B	107.7	C18—C23—H23	120.0
C9—C10—H10A	109.5	C3—N1—C7	122.52 (19)
C9—C10—H10B	109.5	C3—N1—C9	120.85 (19)
H10A—C10—H10B	109.5	C7—N1—C9	116.48 (18)
C9—C10—H10C	109.5	C11—N2—C12	126.83 (19)
H10A—C10—H10C	109.5	C11—N2—H2N	110.9 (15)
H10B—C10—H10C	109.5	C12—N2—H2N	122.3 (16)
N2—C11—C6	123.9 (2)	C18—N3—C15	125.3 (2)
N2—C11—H11	118.0	C18—N3—H3H	114.8 (15)
C6—C11—H11	118.0	C15—N3—H3H	114.7 (16)
			
O1—C1—C2—C3	−179.1 (2)	N2—C12—C17—C16	−178.5 (2)
C6—C1—C2—C3	1.7 (3)	N3—C18—C19—C20	177.1 (2)
C1—C2—C3—N1	−179.8 (2)	C23—C18—C19—C20	−1.3 (3)
C1—C2—C3—C4	−0.3 (3)	C18—C19—C20—C21	0.5 (3)
N1—C3—C4—C5	178.5 (2)	C19—C20—C21—C22	0.4 (3)
C2—C3—C4—C5	−1.0 (3)	C20—C21—C22—C23	−0.5 (3)
C3—C4—C5—C6	0.8 (3)	C21—C22—C23—C18	−0.4 (3)
C4—C5—C6—C11	−178.0 (2)	N3—C18—C23—C22	−177.1 (2)
C4—C5—C6—C1	0.8 (3)	C19—C18—C23—C22	1.2 (3)
O1—C1—C6—C11	−2.4 (3)	C2—C3—N1—C7	−175.9 (2)
C2—C1—C6—C11	176.8 (2)	C4—C3—N1—C7	4.5 (3)
O1—C1—C6—C5	178.8 (2)	C2—C3—N1—C9	−0.5 (3)
C2—C1—C6—C5	−2.0 (3)	C4—C3—N1—C9	180.0 (2)
C5—C6—C11—N2	177.8 (2)	C8—C7—N1—C3	77.6 (3)
C1—C6—C11—N2	−0.9 (3)	C8—C7—N1—C9	−98.1 (2)
C17—C12—C13—C14	−3.6 (3)	C10—C9—N1—C3	−84.0 (3)
N2—C12—C13—C14	177.3 (2)	C10—C9—N1—C7	91.7 (3)
C12—C13—C14—C15	1.3 (3)	C6—C11—N2—C12	−178.3 (2)
C13—C14—C15—C16	2.2 (3)	C17—C12—N2—C11	160.3 (2)
C13—C14—C15—N3	179.0 (2)	C13—C12—N2—C11	−20.5 (3)
C14—C15—C16—C17	−3.4 (3)	C23—C18—N3—C15	1.5 (3)
N3—C15—C16—C17	179.9 (2)	C19—C18—N3—C15	−176.8 (2)
C15—C16—C17—C12	1.2 (4)	C14—C15—N3—C18	127.6 (2)
C13—C12—C17—C16	2.3 (3)	C16—C15—N3—C18	−55.7 (3)

### Hydrogen-bond geometry (Å, º)

Cg is the centroid of the C1–C6 ring.

**Table:**

*D*—H···*A*	*D*—H	H···*A*	*D*···*A*	*D*—H···*A*
N2—H2*N*···O1	0.90 (2)	1.83 (2)	2.609 (2)	143 (2)
N3—H3*H*···O1^i^	0.85 (2)	2.05 (2)	2.900 (3)	175 (2)
C7—H7*A*···*Cg*^ii^	0.97	2.87	3.465 (3)	121

Symmetry codes: (i) −*x*, *y*+1/2, −*z*+1/2; (ii) *x*, −*y*+1/2, *z*−1/2.
